# The Central Paratethys Sea—rise and demise of a Miocene European marine biodiversity hotspot

**DOI:** 10.1038/s41598-024-67370-6

**Published:** 2024-07-15

**Authors:** Mathias Harzhauser, Bernard Landau, Oleg Mandic, Thomas A. Neubauer

**Affiliations:** 1 Natural History Museum Vienna, Burgring 7, 1010 Vienna, Austria; 2 Institut Für Erdwissenschaften, NAWI Graz Geocenter, Universität Graz, Heinrichstraße 26, 8010 Graz, Austria; 3 Instituto Dom Luiz da Universidade de Lisboa, Campo Grande, 1749-016 Lisboa, Portugal; 4 International Health Centres, Av. Infante de Henrique 7, Areias São João, P-8200 Albufeira, Portugal; 5 SNSB—Bavarian State Collection for Paleontology and Geology, Richard-Wagner-Straße 10, 80333 Munich, Germany; 6 Naturalis Biodiversity Center, P.O. Box 9517, 2300 RA Leiden, The Netherlands

**Keywords:** Miocene climatic optimum, Middle Miocene climate transition, Biodiversity hotspot, Paratethys Sea, Gastropoda, Palaeoceanography, Palaeoclimate

## Abstract

The Miocene Climate Optimum (MCO, ~ 17–14 Ma) was a time of extraordinary marine biodiversity in the Circum-Mediterranean Region. This boom is best recorded in the deposits of the vanished Central Paratethys Sea, which covered large parts of central to southeastern Europe. This sea harbored an extraordinary tropical to subtropical biotic diversity. Here, we present a georeferenced dataset of 859 gastropod species and discuss geodynamics and climate as the main drivers to explain the changes in diversity. The tectonic reorganization around the Early/Middle Miocene boundary resulted in the formation of an archipelago-like landscape and favorable conditions of the MCO allowed the establishment of coral reefs. Both factors increased habitat heterogeneity, which boosted species richness. The subsequent cooling during the Middle Miocene Climate Transition (~ 14–13 Ma) caused a drastic decline in biodiversity of about 67%. Among the most severely hit groups were corallivorous gastropods, reflecting the loss of coral reefs. Deep-water faunas experienced a loss by 57% of the species due to changing patterns in circulation. The low sea level led to a biogeographic fragmentation reflected in higher turnover rates. The largest turnover occurred with the onset of the Sarmatian when bottom water dysoxia eradicated the deep-water fauna whilst surface waters-dwelling planktotrophic species underwent a crisis.

## Introduction

**Figure 1:**
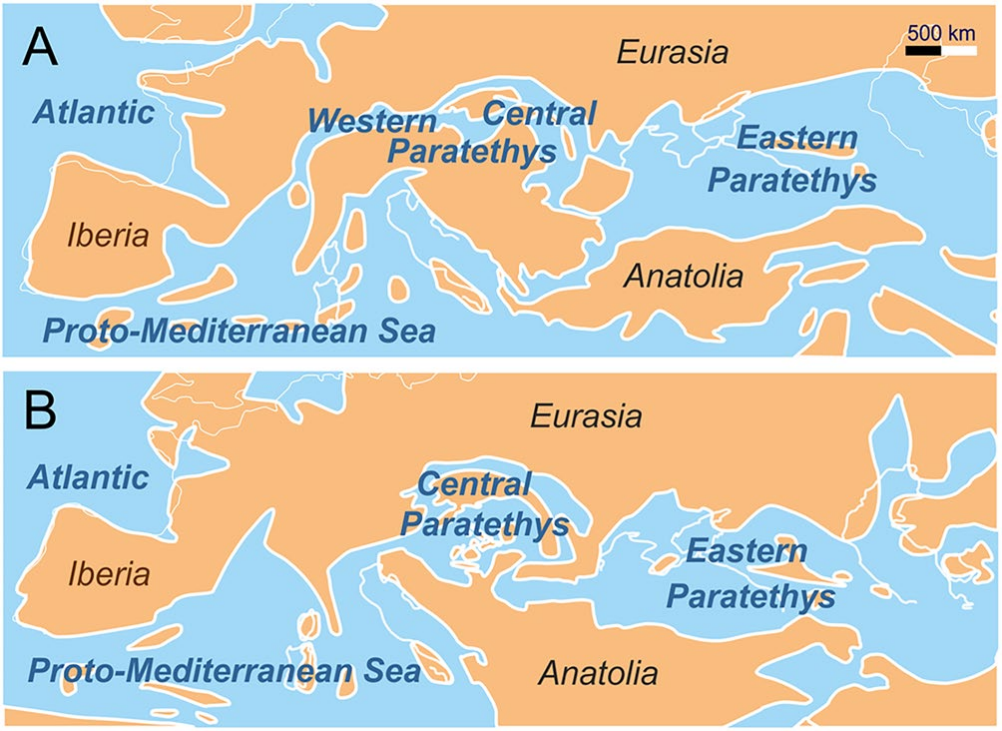
Paleogeography of the Circum-Mediterranean Region during the Burdigalian (**A)** and Langhian (**B)** (modified from Popov et al.^4^). Maps created with CorelDRAW 2019, https://www.coreldraw.com/.

**Figure 2:**
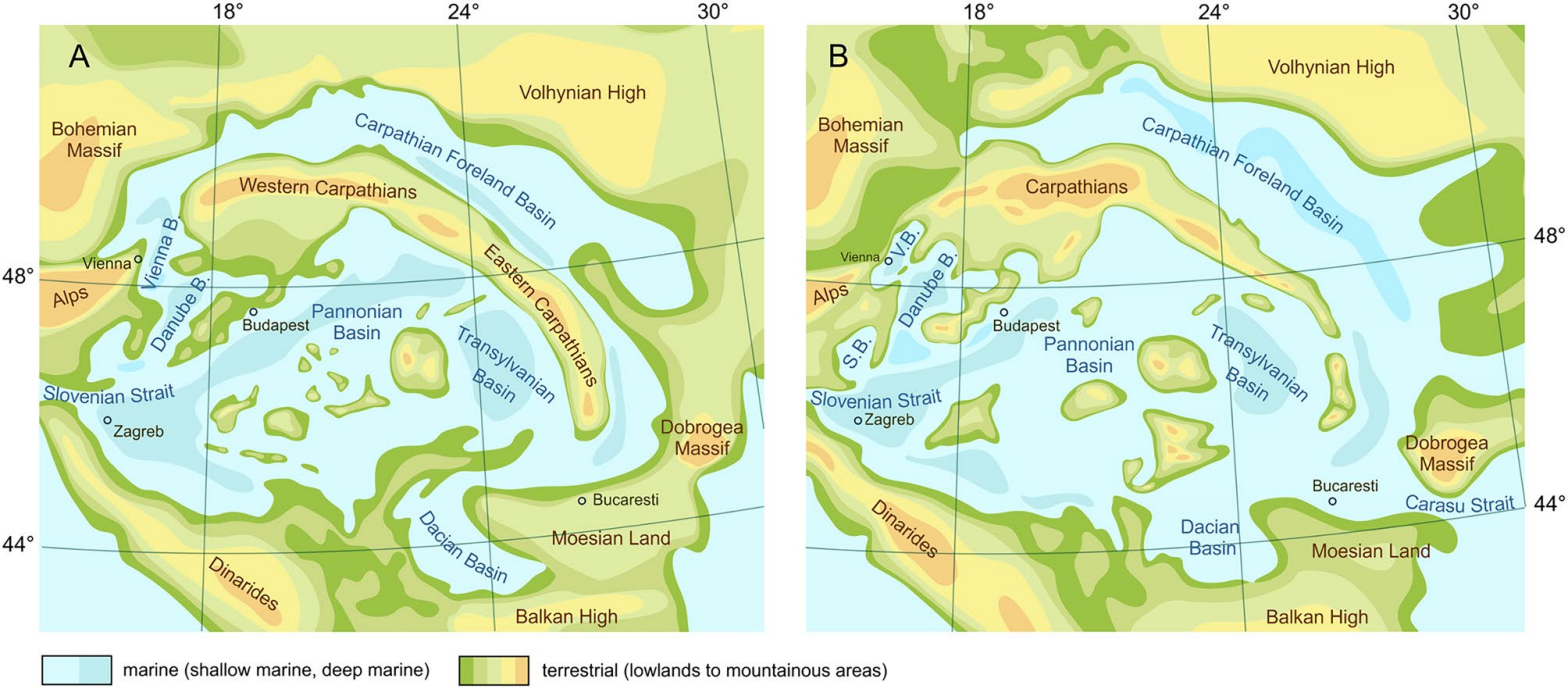
Paleogeographic situation of the Central Paratethys Sea during the Langhian/early Badenian (**A**) and the Serravallian/late Badenian (**B**) with major sedimentary basins and gateways to adjacent regions (modified from Popov et al.^4^). Maps created with CorelDRAW 2019, https://www.coreldraw.com/.

The Paratethys Sea was a huge epicontinental sea that came into existence around the Eocene/Oligocene boundary due to the emerging mountain ranges, which divided the former Tethys Ocean during the Alpine orogeny. Since Laskarev^1^ coined the term Paratethys, numerous papers investigated the evolution of this sea^2–6^. The most influential maps, which are still widely in use, were published by Rögl^2^ and Popov et al.^4^. Popov’s maps are also used herein as base for our reconstructions (Figs. 1 and 2).

**Figure 3:**
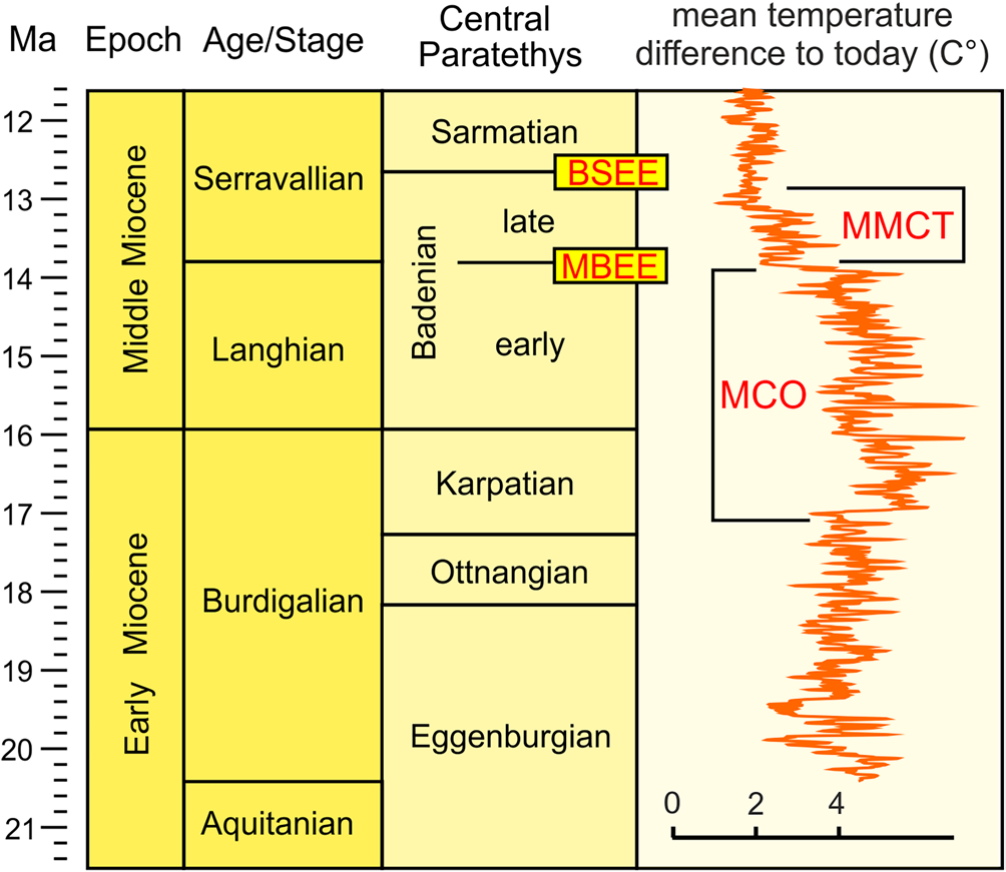
Stratigraphic table for the Early and Middle Miocene showing the correlation of the regional stages of the Central Paratethys with international stratigraphic units. The climate curve indicates the position of the Miocene Climatic Optimum (MCO) and Middle Miocene Climate Transition (MMCT) after Westerhold et al.^30^. International stratigraphic stages modified from Gradstein et al.^20^. MBEE = Mid-Badenian Extinction Event, BSEE = Badenian/Sarmatian Extinction Event.

The Paratethys Sea was divided into three regions. During the Oligocene and Early Miocene, the North-Alpine Foreland Basin was part of the Paratethys Sea sometimes referred to as Western Paratethys^6,7^ (Fig. 1A). The Central Paratethys covered the Pannonian Basin and the Alpine-Carpathian Foreland Basin; here, we focus only on this part of the sea. The Eastern Paratethys ranged from Ukraine to Bulgaria in the west to Kazakhstan and Uzbekistan in the east (Fig. 1)^4,8–11^. The Western Paratethys disappeared during the late Early Miocene, due to tectonic uplift^12^. The Central Paratethys existed until the Middle/Late Miocene boundary, when the area was transformed into the brackish Lake Pannon that gradually became filled by fluvial deltas during the Pliocene^13–15^. The Eastern Paratethys persisted throughout the Oligocene to Pleistocene with strongly changing shorelines, and its remnants are still represented by the Black Sea, the Caspian Sea and the Aral Sea^4,16–18^. Due to its complex paleogeographic history, a system of regional stages was developed for Paratethyan deposits (Fig. 3)^19,20^.

Hundreds of papers have been devoted to the taxonomic description and analysis of Paratethyan fossils, and its biota is reasonably well known. However, surprisingly few attempts have been made to analyze the distribution of selected taxonomic groups on a pan-Paratethyan scale. Studencka et al.^21^ studied the connectiveness of the Paratethyan bivalve faunas and Harzhauser & Piller^5^ focused on the gastropods and foraminifera. These authors utilized literature data and focused on large-scale patterns, such as major faunal turnovers; e.g., the Mid-Badenian Extinction Event (MBEE) at ~ 13.8 Ma and the Badenian/Sarmatian Extinction Event (BSEE) at ~ 12.7 Ma (Fig. 3). Paratethyan scleractinian reef corals were discussed by Perrin & Bosellini^22^, who had a Circum-Mediterranean focus.

All former studies were literature-based data collections, amalgamating a multitude of different taxonomic traditions, incongruent species concepts and a generally broadly scattered quality of research. These issues render many taxonomic datasets internally incomparable and can severely bias reconstructions of species richness, biogeographic relationships and evolutionary trajectories—a problem that is frequently underestimated in (paleo) biodiversity research^23,24^. To overcome this problem and to minimize the impact of taxonomic artefacts we specifically use a dataset that was developed by critical taxonomic revisions performed mainly by M.H. and B.L. during the last 15 years. Our dataset does not include data from unrevised historical literature. It comprises 858 species from 95 localities (Supplementary Fig. 1) of marine gastropods from the Central Paratethys Sea, spanning the entire Early–Middle Miocene and representing one of the largest consistently and critically evaluated paleontological-malacological species-level datasets (for details see Methods). Although our data set covers only a part of the total marine gastropod fauna of the Central Paratethys Sea, it comprises many speciose families of the orders Trochida, Littorinimorpha and Neogastropoda. As such we consider it a representative sample to deduce general patterns.

The concept of a biodiversity hotspot was introduced by Meyers^25^ for areas with great biological diversity and high levels on endemism. For biologists, biodiversity hotspots are crucial for conservation strategies to cope with anthropogenic threat and habitat destruction^26^. To apply also to paleontological datasets Renema et al.^27^ modified the concept slightly and defined biodiversity hotspots as geographic areas with a maximum of diversity in a given time interval. Herein, we follow this approach.

## Results

**Figure 4:**
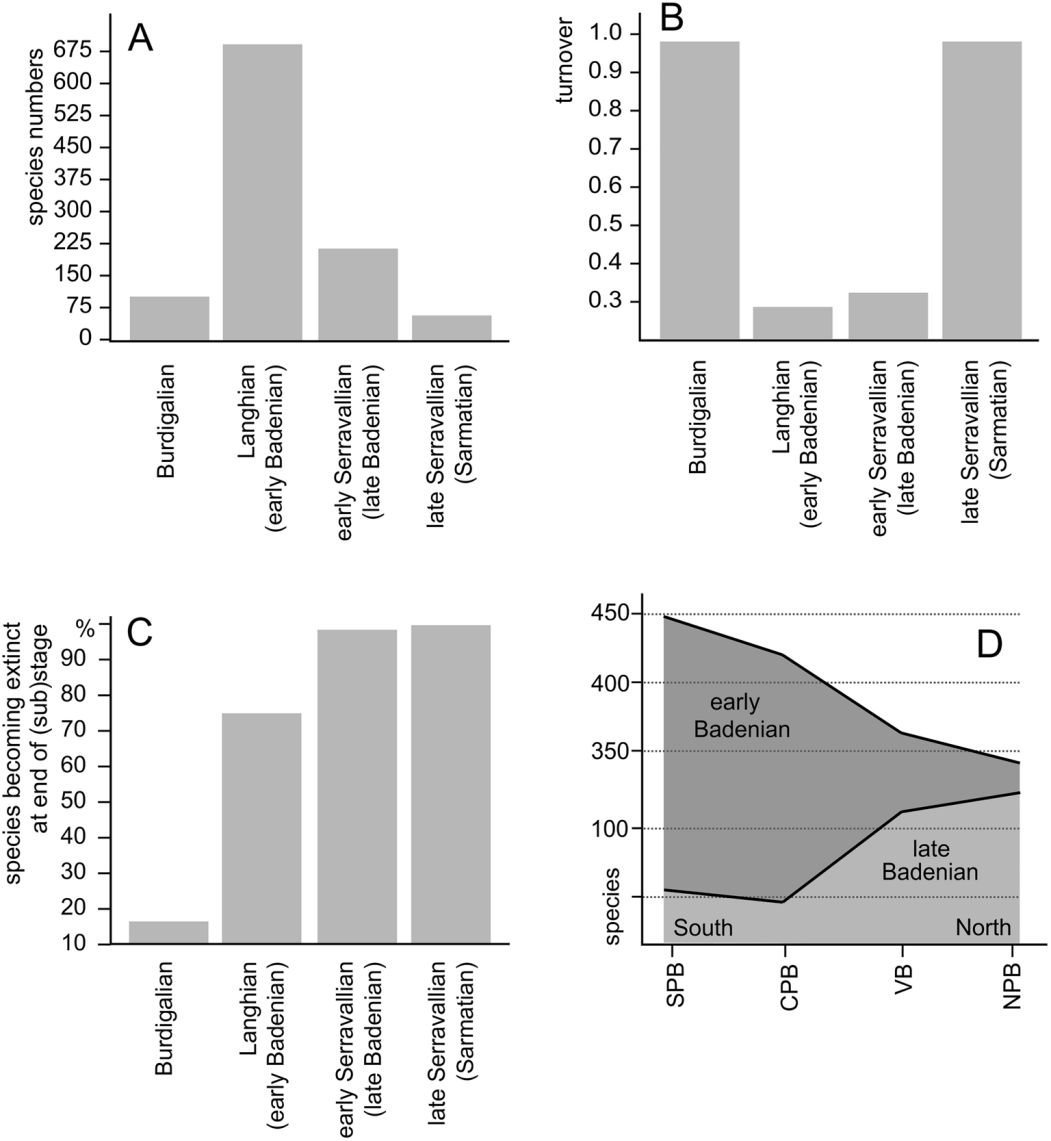
Species richness per interval (**A**), turnover rates between faunas (**B**) and extinctions at (sub) age boundaries (**C**). North–South gradients in species richness for the Langhian and the Serravallian (**D**); NFB: Northern Paratethyan Basin, VB: Vienna Basin, CPB: Central Paratethyan Basin, SPB: Southern Paratethyan Basin. Data for end-Sarmatian extinction from Harzhauser & Piller^5^.

Our data document considerable temporal and spatial differences in diversity of the Paratethyan gastropod faunas. The number of species per (sub)age are 51 for the early Burdigalian (Eggenburgian), 16 for the middle Burdigalian (Ottnangian), 88 for the late Burdigalian (Karpatian), 698 for the Langhian (early Badenian), 237 for the early Serravallian (late Badenian) and 54 for the late Serravallian (Sarmatian) (Fig. 4A). The low species numbers for the Eggenburgian and Ottnangian are almost certainly a result of taphonomic bias due to the generally poor preservation and loss of small species and must be interpreted with caution. Turnover rates between the stages are highest at the Ottnangian/Karpatian boundary and the Badenian/Sarmatian boundary but are low at the Karpatian/Badenian and the early/late Badenian boundaries (Fig. 4B). The high percentage of species persisting from the Early Miocene into the Middle Miocene and from the Langhian into the Serravallian is opposed by increasing extinction rates, which rose from 15.9% at the Early/Middle Miocene boundary to 75.6% at the Langhian/Serravallian boundary and peaked at 98.1% with the onset of the Sarmatian (Fig. 4C).

**Figure 5:**
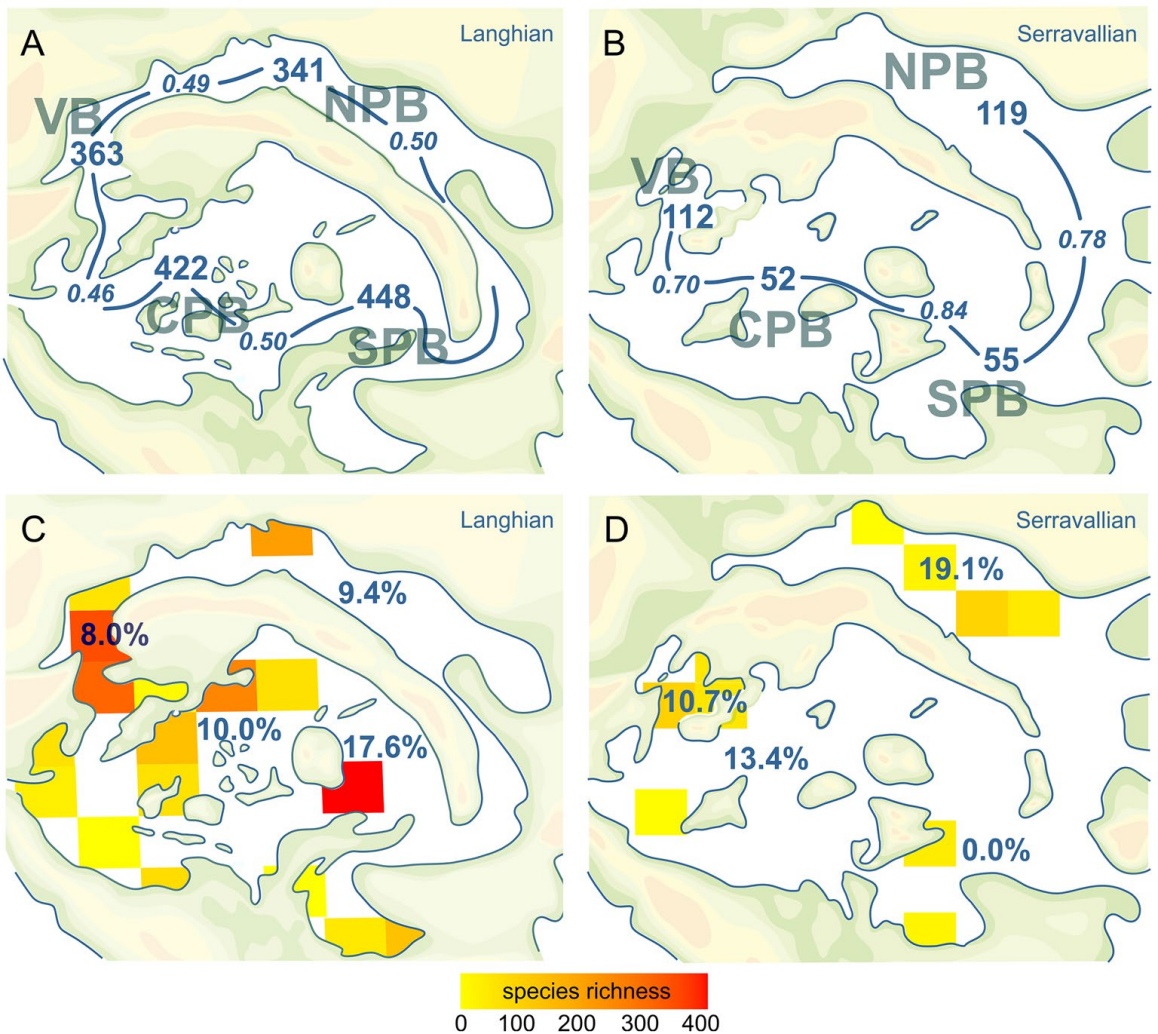
Species numbers, beta diversity (**A**, **B**) and gridded species richness (**C**, **D**) for the Langhian and the Serravallian; values in C and D indicate endemism in %. VB: Vienna Basin, NPB: Northern Paratethyan Basin, CPB: Central Paratethyan Basin, SPB: Southern Paratethyan Basin. Maps created with CorelDRAW 2019, https://www.coreldraw.com/.

The data for the Early Miocene is too sparse to discuss spatial patterns, but the rich Middle Miocene occurrences allow for comparisons between subregions within the Central Paratethys Sea. In the Langhian (early Badenian), species richness decreased from the south to the north (Fig. 4D). The trend reversed in the Serravallian (late Badenian) when the highest diversity is recognized in the Northern Paratethyan Basin (NPB). The changing faunistic similarity between the subregions is expressed by increasing beta diversity among basins. The Jaccard distance ranges between 0.52 and 0.60 for Langhian faunas (Fig. 5A) but increases to 0.79 and 0.90 for Serravallian faunas (Fig. 5B). The larger part of these ranges is a result of differences in species composition as shown by relatively high turnover components (0.46–0.51 for the Langhian, 0.64–0.84 for the Serravallian (Supplementary Table 2). Similarly, the multiple-site dissimilarity and its turnover component were distinctly higher for the Serravallian (total: 0.88, turnover: 0.82) than for the Langhian (total: 0.67, turnover: 0.62).

The number of endemic species per subregion is rather uniform during the Langhian, in the NPB, VB and CPB, ranging from 8 to 10% but increases strongly in the SPB, where 17.6% of the species are not known so far from other regions (Fig. 5C). During the Serravallian, endemism is highest in the NPB (19.1%) and ranges from 10.7 to 13.4% in the VB and the CPB (Fig. 5D). No endemics are known from the SPB. The increasing biogeographic structuring from the Langhian to the early Serravallian is also expressed by the results of the Mantel test. The test is not significant for the Langhian dataset (r = 0.028, *p* = 0.349) but indicates considerable structure for the early Serravallian (r = 0.449, *p* = 0.002).

The subsampling procedure showed a high match between subsampled and original beta diversity values. Even when using only 40% of the data, the median Spearman’s rank correlation coefficient was over 0.97 (Supplementary Fig. 2). This result shows that the signal is robust and suggests further that the picture is not influenced by the taxon selection and is not expected to change considerably with a larger dataset.

## Discussion

### Tectonics and climate as drivers for the establishment of a diversity hotspot

The Paratethyan gastropod fauna was strongly shaped by geodynamics and global climatic change. Our data show that gastropod species richness in the Central Paratethys Sea increased distinctly from the Early to the Middle Miocene. The timing of this diversity peak correlates with two major events, suggesting a causal relation:

1. Tectonic reorganization transformed the Paratethyan basins from W-E oriented basins to a system of extensional basins^2,6^. Consequently, the paleogeography of the Central Paratethys Sea changed dramatically and an archipelago style landscape arose during the Middle Miocene (Fig. 2). This highly structured paleogeography might have been a key-factor for the striking rise in diversity observed during the Langhian. At that time, the sea was connected with the Proto-Mediterranean Sea via the Slovenian Strait^28^, which allowed for exchange between both seas.
2. Favorable conditions during the Miocene Climatic Optimum^29,30^ caused subtropical to tropical conditions in the Circum-Mediterranean Region and supported the development of a unique diversity hot-spot harboring 699 species with numerous thermophilic elements. Global mean surface temperatures were about 3 to 6°C higher than today^31,32^ and rising sea surface temperatures allowed for the establishment of complex coral reefs in the Central Paratethys^22,33,34^. Our data corroborate the models proposed by Leprieur et al.^35^, who showed that tectonics played a major role in reef biodiversity since the Late Cretaceous. The establishment of reefs is also reflected by a strong increase of coral-associated gastropods (e.g., Architectonicidae, Coralliophilinae, Mathildidae). Again, this pattern agrees with observations in the modern IWP region that high biodiversity correlates with the availability of reef-associated shallow-water habitats^36^. We hypothesize that the fortunate coincidence of tectonics and climate warming led to an increase in habitat heterogeneity, which stimulated the formation of a diversity hotspot. The heterogeneity is also expressed by a moderately high beta diversity pointing to considerable differences between local faunas.

The hotspot was established by an overall increase in diversity of all families studied. Nevertheless, the most speciose families were the Muricidae (112), Conidae (71), Clavatulidae (62), Nassariidae (54), Cancellariidae (44), Mitridae (35), Columbellidae (34) and Costellariidae (32) (species numbers in brackets). This clearly indicates that the early Middle Miocene diversity hotspot was essentially developed by Neogastropoda. This is in line with molecular phylogenies of several Neogastropoda families, which suggest major radiations during the Miocene^37,38^. The early Middle Miocene diversity of the Central Paratethys was 2.5 times higher than that of the modern Mediterranean Sea with only 272 species^39^ and was even higher than that of the modern tropical eastern Atlantic (593 species)^40^ and that of the modern Red Sea (464 species)^41^. Nevertheless, it was far lower than that of the modern Coral Triangle (1504 species)^42^ (species numbers refer only to the families treated herein).

### Reversing diversity gradients

Paleogeographic reconstructions using the GPlates web service (https://gwsdoc.gplates.org/) place the area of Vienna at a paleolatitude of 47.5° during the Langhian and at 47.7° during the Serravallian (today: 48.2°). Therefore, plate tectonic movements were negligible in the time frame studied herein and changes in the composition of the faunas have to be explained by other mechanisms.

The large number of species reveals the Central Paratethys as a marine diversity hotspot during the Langhian. However, species numbers are not uniformly distributed between northern and southern basins. A north–south gradient in species diversity was already discussed by Harzhauser et al.^43^, who observed that some iconic mollusk species were restricted to the southern basins (e.g., the stromboids *Europrotomus schroeckingeri* and *Pereiraea gervaisii*). Similarly, the distribution of coral reefs indicates a separation between southern Paratethyan basins, with complex reefs, and northern basins, lacking such ecosystems^44,45^. According to the data of those authors, the northern limit of the Paratethyan reef belt was situated in a region that ranges nowadays from 46.7° to 47.8°, roughly marking the boundary between the Pannonian Basin and the Vienna Basin.

Our quantitative data confirm this trend and document a gradual decline of diversity of about 23% from a southern diversity hotspot with about 448 species, towards slightly lower diversities in the Northern Paratethyan Basin (NPB) with about 341 species (Figs. 4D and 5C). This hotspot formed around 15 to 14 Ma, when the Miocene Climatic Optimum was at full swing and the Central Paratethys an archipelago with complex coral reefs and high habitat heterogeneity. For the first time, however, we document the reversal of this pattern during the Serravallian (late Badenian). At that time, the NPB harbored the highest diversity of gastropods with about 119 species. Less than half of this number occurred also in the other regions. This striking pattern was caused by endemic radiations in some families, such as the Cerithiidae (own data M.H.), Nassariidae^46^, Muricidae and Costellariidae^47^. The drop in global sea level of about 50 m during the MMCT^48,49^ initiated in the Central Paratethys Sea the Badenian Salinity Crisis^50^ and caused fragmentation of the basins.

This fragmentation did not necessarily result in full geographic isolations between basins but even if marine connections persisted, the faunistic exchange became hampered by shallow sills. For example, a water depth of less than 220 m was enough to separate the Proto-Mediterranean Sea from the Indian Ocean hydrologically^51^.

After the crisis, the Carpathian Foreland Basin became normal saline again^52^. At that time, a second, less speciose Paratethyan diversity hotspot developed in this semi-enclosed basin along the northern margin of the Central Paratethys Sea. Conversely, the former diversity hotspot in the SPB had completely vanished, which is also expressed by a dramatic decrease of endemism from 17.6% to zero (Fig. 5C,D).

### The Miocene climate transition as cause for a major diversity collapse

**Figure 6:**
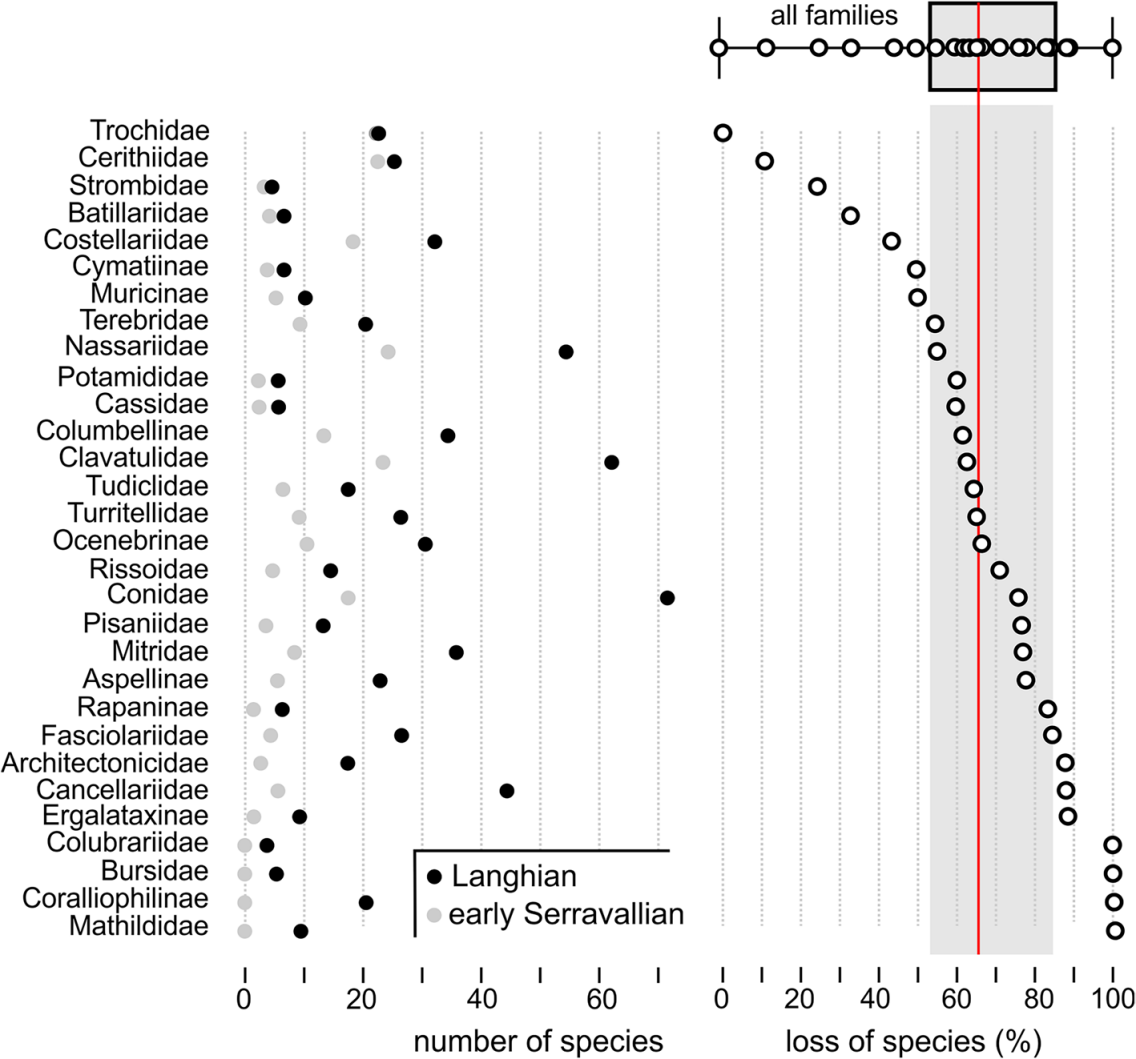
Numbers of species per family for the Langhian (early Badenian) and the early Serravallian (late Badenian) and loss of species per family between the Langhian and Serravallian marking the Mid-Badenian Extinction Event. The red line signals the global mean of the pooled data set; the gray bar represents the 25th and 75th percentiles of the boxplot.

The boundary between the Langhian and Serravallian falls within the onset of the Middle Miocene Climatic Transition (MMCT) (Fig. 3). This global climatic event is reflected by the expansion of Antarctic ice sheets^30,53^ and a drop of the global sea level of about 50 m^48,49^. In the Paratethyan gastropod faunas the MMCT is reflected by a major drop in species numbers. This event was coined Middle Badenian Extinction Event (MBEE) by Harzhauser & Piller^5^, who, however, were not able to reliably quantify the magnitude of this event. Data collected by our team since 2007 show that on average, the Serravallian fauna was 67.2% poorer in species compared to the highly diverse Langhian fauna. However, gastropod families were not uniformly affected by the MBEE (Fig. 6). Intertidal and coastal groups, such as Trochidae, Cerithiidae and Batillariidae, were least affected and display comparable species diversity before and after the event (note that these group are rare or absent in deep water settings in the Paratethys Sea). Species of families dwelling preferably in shallow to medium deep sublittoral environments were strongly reduced, typically ranging between 57.5 and 83.4% (= 25th and 75th percentiles in boxplot of Fig. 6). High losses of species are observed in Architectonicidae, Coralliophilinae and Mathildidae, which feed on coelenterates^54–56^. This decline parallels the decline of coral reefs in the Central Paratethys during the MMCT^34,44^. The marked drop in species numbers in Bursidae, Cancellariidae, Fasciolariidae and Rapaninae, which are less prey-sensitive, might be linked to lowered temperatures, because many species of these families are thermophilic^57^. The fragmentation of the Serravallian fauna is indicated by a drastic increase in the beta diversity (Fig. 5A,B).

### A zooplankton crisis in surface waters

Most shells of marine gastropods conserve the early ontogeny of a species by the morphology of its protoconch. Planktotrophic larvae with a long planktonic stage typically have multispiral protoconchs, whereas non-planktotrophic species, which are either directly developing or have a short lecithotrophic pelagic phase, have paucispiral protoconchs^58,59^. Although there exists no clear cut-off point between both modes, protoconchs with a large nucleus and less than 2.25 whorls suggest lecithotrophic development and protoconchs with a small nucleus and more than three whorls suggest planktotrophic development^59^). As a rule, species with long planktotrophic life history are more likely to disperse widely and are geologically long-lived. In contrast, species with short larval phases are less widely distributed and tend to form genetically isolated populations leading to species that are geographically restricted and geologically short-lived^59,60^. Such endemic radiations of non-planktotrophic gastropods are documented for example for extant *Euthria* (Tudiclidae) and *Lautoconus* (Conidae) in the Cape Verde Archipelago^61,62^.

**Figure 7:**
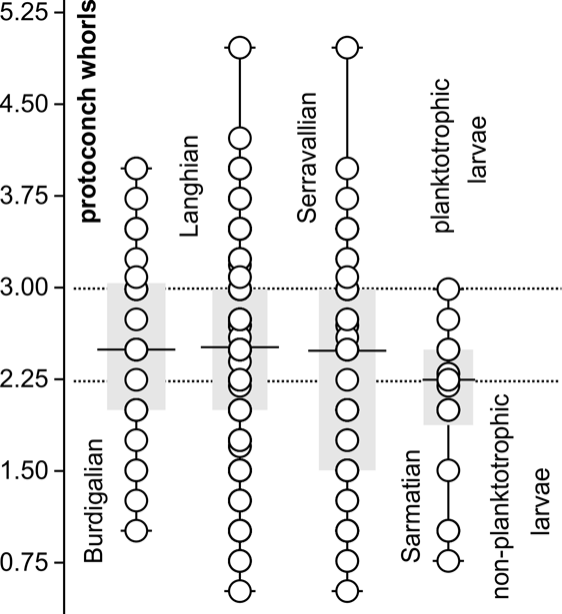
Number of protoconch whorls per species and interval; dotted lines represent boundaries between planktotrophic and non-planktotrophic larval types; interval between may comprise both types.

During the Langhian, Paratethyan surface waters were connected to the Proto-Mediterraean Sea via strong current systems and upwelling systems were wide-spread^63,64^. This situation favored planktotrophy. In contrast, the fragmentation of the Paratethyan basins and the isolation of individual basins during the sea-level low of the MMCT might be expected to have favored endemic radiations of non-planktotrophic species. This hypothesis would explain the observed early Serravallian (late Badenian) diversity hotspot in the NPB. However, the average number of protoconch whorls did not change from the Early Miocene throughout the Langhian and (pre-Sarmatian) Serravallian (Fig. 7). The predominance of planktotrophic larval types persisted from the MCO through the MMCT. The reason for this might have been the increase in productivity in Paratethyan surface waters during the Serravallian^63,65,66^, which supported planktotrophy. Similarly, Landau et al.^67^ documented that productivity promoted planktotrophic development in the Caribbean Neogene. A distinct shift towards non-planktotrophic larval development occurred after the Badenian–Sarmatian Extinction Event (BSEE) (Fig. 7). This shift was especially prominent within Nassariidae^46^, suggesting an evolutionary advantage for direct developers in the Sarmatian Sea and unfavorable conditions for planktonic larvae. This observation is in line with the near complete break-down of planktic foraminiferal communities at the BSEE^5,68^. It seems that surface waters became hostile for large parts of the zooplankton during the early Sarmatian. The causes for this collapse of the zooplankton are so far unknown. The amplitude of the BSEE can at least partly be explained by this crisis in planktotrophs.

### The benthic deep-water fauna as victim of changing circulation patterns

**Figure 8:**
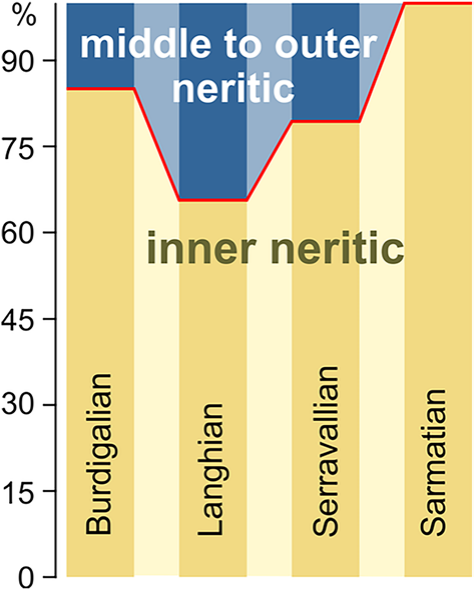
Ratio between the numbers of shallow-water species versus species from deep-water environments.

**Figure 9:**
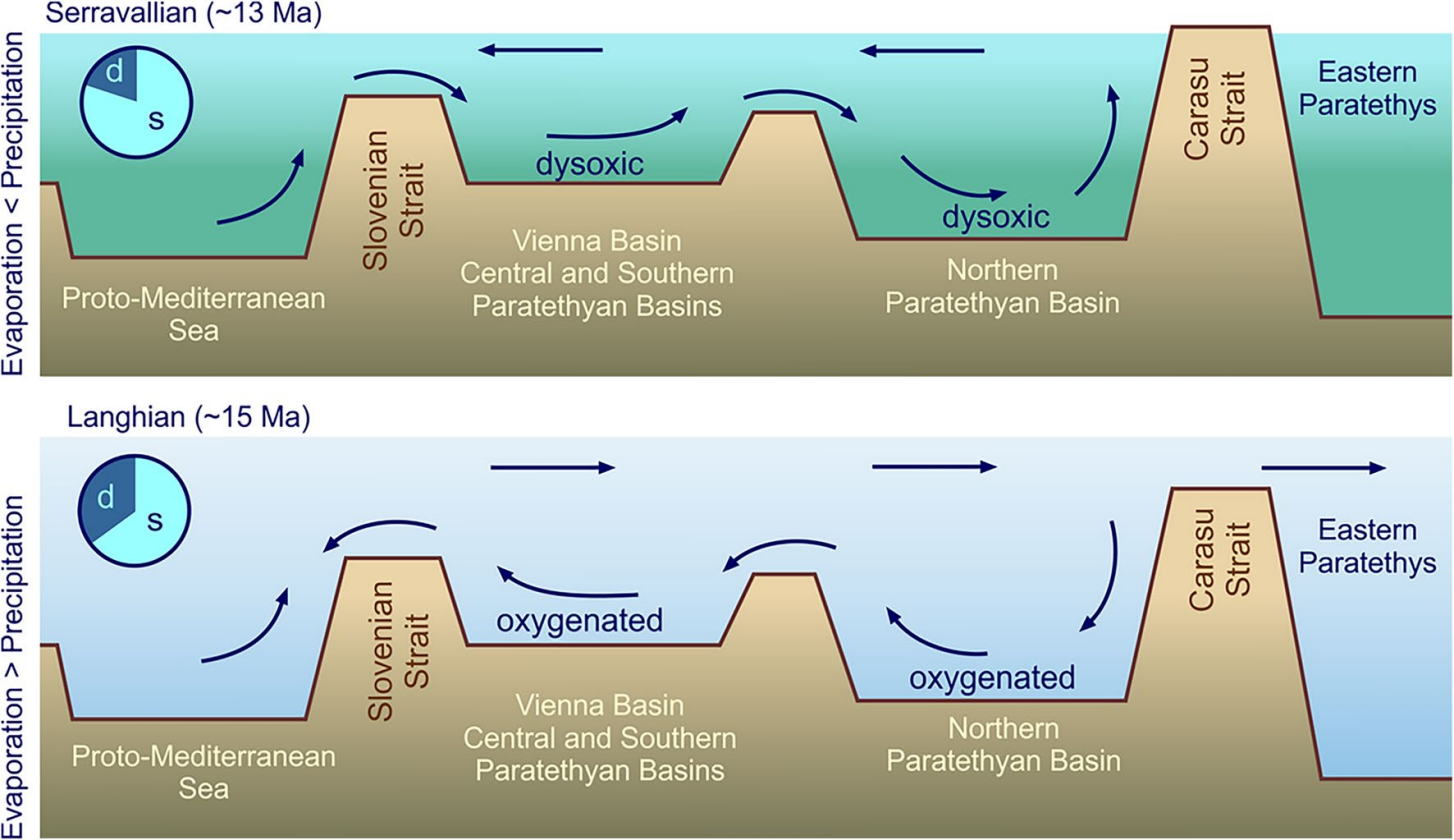
The change from an antiestuarine circulation during the Langhian towards an estuarine circulation during the Serravallian had a major impact of the deep-water fauna; circles: ratio between deep-water species (d) and shallow-water species (s). Circulation model modified from Báldi^72^, topography modified from Sant et al.^69^.

The Paratethyan fossil record is strongly dominated by species from inner neritic, coastal marine environments. The number of species from shallow-marine settings ranged from 65% during the Langhian, to 80–85% during the Burdigalian and Serravallian and peaked at 100% in the Sarmatian (Fig. 8). The highest number of deep-water species was reached during the Langhian, when the Langhian flooding is reflected by widespread offshore clays throughout the Central Paratethys^69,70^. The marine gastropod fauna of these deposits was not only rich in species, but some Turridae and Naticidae occurred in enormous numbers as witnessed by tens of thousands of specimens in the paleontological collections of the region (M.H., pers. observ.). The diversity and abundance of the deep-water fauna declined by 57% from the Langhian to the Serravallian, although offshore clays were still widespread in the Central Paratethys. An explanation for this surprising pattern is the tectonically and climatically induced change from an antiestuarine circulation during the Langhian^71^ to an estuarine circulation pattern in the Serravallian^72^ (Fig. 9). This change resulted in the formation of widespread bottom water dysoxia, as reflected especially by the benthic foraminiferal assemblages^64,73,74^. The resulting poorly oxygenated deep-water environments of the Serravallian were unfavorable for many gastropods. The incipient Serravallian decline of deep-water species culminated in a complete break-down of the offshore gastropod fauna with the onset of the Sarmatian. Again, widespread dysoxia established in basinal settings^75,76^. Sarmatian offshore mollusk faunas were dominated by bivalves such as *Abra* and thin-shelled cardiids, which seemingly were adapted to this poorly oxygenated environment^76^. The loss of deep-water species was an additional factor explaining the large magnitude of the BSEE.

## Conclusions

Our collected, taxonomically revised species occurrence dataset reveals that the Central Paratethys Sea formed an outstanding marine gastropod species richness hotspot during the Middle Miocene after a phase of low species numbers during the Early Miocene. Although we note that the Early Miocene paucity of species may in part be due to taphonomic bias. The warm climate of the MCO caused a major flooding, which covered vast areas of the Central Paratethyan basins. At around 15–14 Ma, an extraordinary diversity hotspot was established in the Southern Paratethyan Basin from where the diversity slightly declined towards the north. Endemism was higher within the diversity hotspot but lower and uniform in other subregions (Fig. 5). The driving forces behind the Langhian/early Badenian peak were favorable climatic conditions and geodynamics, which formed an archipelago-like landscape. Both factors increased habitat heterogeneity, as witnessed by the appearance of complex coral reefs at that time. This heterogeneity is also expressed by a moderately high beta diversity between the subregions.

The lowered sea level during the subsequent MMCT led to a fragmentation of the Paratethyan basins. Gastropod species numbers declined considerably, and the surviving fauna was strongly disconnected. Consequently, the beta diversity strongly rose between all subregions. The southern diversity hotspot vanished and instead a less speciose hotspot developed in the Northern Paratethyan Basins, fed by local radiations. The changing hydrological setting resulted in the collapse of the prevailing antiestuarine circulation pattern, and the Serravallian estuarine circulation system caused widespread bottom water dysoxia (Fig. 9). This hydrological change is reflected by a dramatic decrease of deep-water faunas. Three quarters of the Langhian species became extinct during the MBEE, but the survivors formed the stock of the Serravallian fauna resulting in comparatively low turnover rates at the Langhian/Serravallian boundary. The most severe faunal turnover occurred slightly later with the onset of the Sarmatian when almost the entire Serravallian gastropod fauna became extinct. Our data suggest that this Badenian/Sarmatian Extinction Event acted via two, so far overlooked, mechanisms. A shift towards non-planktotrophic larvae points to a severe crisis for zooplankton in the Paratethyan surface waters. Simultaneously, the deep-water gastropod fauna was severely reduced by the formation of dysoxic bottom water conditions. This implies that geodynamics played a major role in the formation of the Langhian diversity hotspot, in the diversity decline during the Serravallian and for the BSEE. In all cases geodynamic effects were amplified by the prevailing climate state. Although strong tectonic reorganizations started at the Early/Middle Miocene boundary, turnover rates remained low at that time. Late Burdigalian and Langhian faunas flourished during the MCO, during which the favorable climatic regime seems to have outpaced the geodynamic impact on these faunas.

The Paratethyan hotspot was geologically short-lived with a duration of no more than 3 Myr. This contrasts with modern biodiversity hotspots, such as the Coral Triangle, which have a considerable geological legacy^77^. The biodiversity hotspot of the Coral Triangle is not only 20 times larger than the Miocene Paratethyan hotspot, but also did not suffer from major extinction events during the Cenozoic, allowing for strong, uninterrupted long-term diversification^77^. Therefore, the peculiar geodynamic situation of the Central Paratethys Sea precluded the establishment of a stable centre of biodiversity. In this sense it was a ‘failed hotspot’.

Concluding, we consider the Central Paratethys Sea a text-book example for the influence of extrinsic factors, such as climate and geodynamics, on the evolution of marine faunas.

## Methods

### Data collection

Following revisions are included: Trochoidea: Trochidae (54 species)^78^; Cerithioidea: Potamididae (9 species)^79^, Batillariidae (11 species)^79^, Turritellidae (37 species)^80–82^, Pickworthiidae (9 species)^83^; Campaniloidea: Plesiotrochidae (4 species) (own data M.H. in prep.); Buccinoidea: Columbellidae (38 species)^84^, Colubrariidae (4 species), Melongenidae (1 species), Pisaniidae (14 species), Prodotiidae (4 species), Tudiclidae (18 species)^85^, Dolicholatiridae (1 species), Fasciolariidae (29 species)^86^, Nassariidae (75 species)^46,87^; Rissooidea: Rissoidae (26 species)^88^; Stromboidea: Strombidae (6 species), Thersitidae (1 species)^89–92^, Tonnoidea: Lauberinidae (1 species)^93^, Tonnidae (2 species), Cassidae (8 species), Ranellidae (1 species), Cymatiidae (7 species), Bursidae (6 species)^94^; Mitroidea: Mitridae (35 species^95^; Conoidea: Terebridae (22 species)^96^, Clavatulidae (79 species)^97^, Conidae (74 species)^98^; Turbinelloidea: Costellariidae (38 species)^47^; Volutoidea: Cancellariidae (54 species)^99,100^; Mathildoidea: Mathildidae (9 species)^101^; Architectonicoidea: Architectonicidae (18 species)^101^; Siphonarioidea: Siphonariidae (2 species)^102^ (Supplementary Table 1). Papers on revised data on Cerithiidae (36 species) and Muricidae (122 species) are under preparation or submitted by our team. New species of these families are given in in the format “*Genus* nov. sp.” with consecutive numbers to avoid nomina nuda.

Despite this wealth of information, 79 families are not included in our dataset, because they have not been revised so far. These families were recorded by Harzhauser & Piller^5^ in their uncritical literature survey and comprise another > 500 species. We still believe that our dataset is a representative sample of the Central Paratethyan gastropod fauna, not only because we have revised species across most of the major clades representing various trophic guilds and habitat preferences. Nonetheless, to ascertain that the effects we infer are unbiased we employ a subsampling approach (see below).

### Geographic occurrence data and stratigraphic coverage

Geographic occurrence data derived exclusively from the critical reevaluations cited above. In total we selected 95 georeferenced localities in Austria, Bosnia and Herzegovina, Bulgaria, Croatia, Czech Republic, Germany, Hungary, Romania, Poland, Serbia, Slovakia, Slovenia and Ukraine (Fig. 2B, Supplementary Fig. 1, Supplementary Table 1). These occurrences cover all major Paratethyan basins and are grouped according to their tectonic setting into the Northern Paratethyan Basin (NPB) (comprising occurrences in the Carpathian Foreland Basin), the Vienna Basin (VB), the Central Paratethyan Basin (CPB) (containing occurrences in the Austrian and Hungarian parts of the Pannonian Basin) and the Southern Paratethyan Basin (SPB), uniting localities in the southern Pannonian Basin, the Transylvanian Basin and Dacian Basin. Stratigraphically, the localities range from the Early to the Middle Miocene and are grouped into six time slices: ~ 21–18.1 Ma, Eggenburgian (early Burdigalian), 18.1–17.2 Ma, Ottnangian (middle Burdigalian), 17.2–16.0 Ma, Karpatian (late Burdigalian), 16.0–13.8 Ma, early Badenian (Langhian), 13.8–12.7 Ma, late Badenian (early Serravallian), 12.7–11.6 Ma, Sarmatian (late Serravallian). For the sake of readability for an audience less familiar with the regional stages, we mainly use international stratigraphic intervals.

### Protoconch type, bathymetry and habitat preference

Protoconch morphology (number of whorls) was evaluated for 294 species (34%) in order to categorize the species as paucispiral (directly developing or lecithotrophic) or as multispiral (planktotrophic). Protoconch whorls are counted including the nucleus, which is the first half whorl. Each species was also categorized according to its presumed bathymetric preference, based on geological context and/or on habitat preferences of extant congeners. This information is available from the revisions listed above.

We treat species occurring in inner neritic coastal settings as shallow water species and species from middle to outer neritic settings as deep water species. The data on the ecological preferences of the species are provided in the systematic papers listed above.

### Statistical analyses

All statistical analyses were carried out in R v. 4.3.2^103^. Gridded species richness maps were created for the Langhian/early Badenian and early Serravallian/late Badenian to illustrate the distribution of species richness and identify centers of diversity. Species occurrences were pooled over a grid of 100 × 100 km in an Equidistant Conic projection with a meridian of 20° and parallels at 32° and 64°, to approximate the paleogeographic maps of Popov et al.^4^. The gridded species richness maps as well as the locality map (Fig. 2B) were created with packages rnaturalearth v. 1.0.1^104^, sf v. 1.0–16^105,106^, sp v. 2.1–3^107,108^, elevatr 0.99.0^109^, raster v. 3.6–26^110^ and ggplot2 v. 3.5.0^111^.

To assess the biogeographic structure of the dataset we calculated beta diversity among time intervals (Eggenburgian/early Burdigalian, Ottnangian/middle Burdigalian, Karpatian/late Burdigalian, Langhian/early Badenian, early Serravallian/late Badenian, late Serravallian/Sarmatian) as well as among basins for the Langhian and early Serravallian subsets. To account for the unevenness of species numbers among time intervals we partitioned beta diversity into two independent components, i.e., spatial turnover, relating to differences in species composition, and nestedness, accounting for differences in species numbers^112^. Here, we focus primarily on the turnover component. Beta diversity and its components were computed using the Jaccard distance with the package betapart v. 1.6^113^. For the early and late Badenian, we additionally computed multiple-site dissimilarity and turnover to assess and compare the degrees of overall spatial heterogeneity in those two time intervals.

Furthermore, a Mantel test was conducted to assess whether taxonomic distances among localities for the Langhian and early Serravallian subsets matches geographic distances. Localities with less than five taxa were excluded from the analyses. Distance matrices were generated with packages vegan v. 2.6–4 ^114^ and geosphere v. 1.5–18^115^, using Jaccard distances for the taxonomic dataset and Haversine distances among localities. The Mantel test was computed using Spearman’s rank correlation coefficient and 9999 permutations.

In order to make sure that the patterns we observed are not a (partial) function of the dataset not containing the entire Central Paratethyan gastropod fauna, we used a subsampling procedure on the beta diversity results. We extracted randomly between 40 and 80% (at an increment of 10%, each based on 999 permutations) of the species and re-calculated all pairwise beta diversity values, between all time intervals in general as well as between basins for the Langhian/early Badenian and early Serravallian/late Badenian. Subsequently, we ran correlation tests between each subsample and the original values, using Spearman’s rank correlation coefficient because the beta diversity values are not normally distributed.

The R code will be made available upon request.

### Supplementary Information

Supplementary Information 1.

Supplementary Information 2.

Supplementary Information 3.

Supplementary Information 4.

Supplementary Information 5.

## Data Availability

The data used are available via the data repository of the Natural History Museum Vienna: 10.57756/mvpks3.
