# Supplementary material for: The Central Paratethys Sea—rise and demise of a Miocene European marine biodiversity hotspot
Source: Sci Rep. 2024 Jul 15;14:16288. doi: 10.1038/s41598-024-67370-6 (PMC11250865; doi:10.1038/s41598-024-67370-6)
Supplement: Supplementary file 1 — Supplementary Information 1. [file 41598_2024_67370_MOESM1_ESM.docx]

**Supplementary Table 1.** List of Gastropoda species with georeferenced occurrence data, number of protoconch whorls (u = unknown) and bathymetric distribution. The dataset is permanently available via the repository of the Natural History Museum Vienna: https://doi.org/10.57756/mvpks3

**Supplementary Table 2.** Results of the beta diversity analyses. Given are total values as well as turnover/nestedness components (all based on Jaccard distance) between all time intervals as well as among basins for the early/late Badenian.

**Supplementary Figure 1.** Geographic setting of the study area with localities (for coordinates and names see Supplementary Table 1). Maps created with CorelDRAW 2019, https://www.coreldraw.com/

**Supplementary Figure 2.** Result of the subsampling approach. Shown are boxplots for Spearman's rank correlation coefficients of all correlation tests between original pairwise beta diversity values and those based on subsampled datasets. Subsampling was carried out across five subsampling ratios using between 40% and 80% of the data, each based on 999 permutations; each correlation is based on 86 beta diversity pairs (self-comparisons were excluded).
