# Supplementary figures and images for: The Central Paratethys Sea—rise and demise of a Miocene European marine biodiversity hotspot
Source: Sci Rep. 2024 Jul 15;14:16288. doi: 10.1038/s41598-024-67370-6 (PMC11250865; doi:10.1038/s41598-024-67370-6)

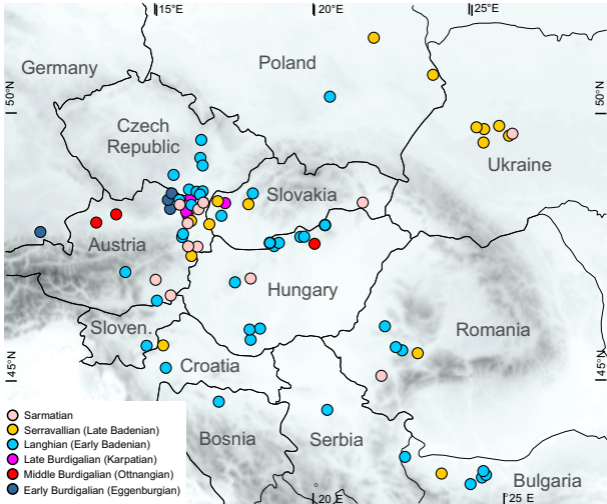

Supplement: Supplementary file 2 — Supplementary Information 2. [file 41598_2024_67370_MOESM2_ESM.pdf]

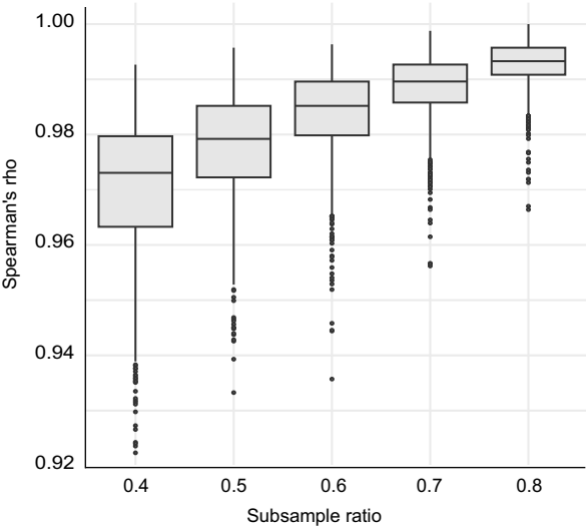

Supplement: Supplementary file 3 — Supplementary Information 3. [file 41598_2024_67370_MOESM3_ESM.pdf]
