# Supplementary material for: The Central Paratethys Sea—rise and demise of a Miocene European marine biodiversity hotspot
Source: Sci Rep. 2024 Jul 15;14:16288. doi: 10.1038/s41598-024-67370-6 (PMC11250865; doi:10.1038/s41598-024-67370-6)
Supplement: Supplementary file 5 — Supplementary Information 5. [file 41598_2024_67370_MOESM5_ESM.pdf]

Beta diversity per time interval and basin

Time interval/Basin1	Time interval/Basin2	Beta div. total	Turnover comp.
Eggenburgian	Ottnangian	0,92	0,81
Eggenburgian	Karpatian	0,91	0,88
Eggenburgian	Early Badenian	0,98	0,87
Eggenburgian	Late Badenian	0,98	0,94
Eggenburgian	Sarmatian	0,99	0,99
Ottnangian	Eggenburgian	0,92	0,81
Ottnangian	Karpatian	0,99	0,97
Ottnangian	Early Badenian	1,00	0,97
Ottnangian	Late Badenian	1,00	0,97
Ottnangian	Sarmatian	1,00	1,00
Karpatian	Eggenburgian	0,91	0,88
Karpatian	Ottnangian	0,99	0,97
Karpatian	Early Badenian	0,90	0,29
Karpatian	Late Badenian	0,85	0,69
Karpatian	Sarmatian	0,98	0,98
Early Badenian	Eggenburgian	0,98	0,87
Early Badenian	Ottnangian	1,00	0,97
Early Badenian	Karpatian	0,90	0,29
Early Badenian	Late Badenian	0,74	0,33
Early Badenian	Sarmatian	0,99	0,93
Late Badenian	Eggenburgian	0,98	0,94
Late Badenian	Ottnangian	1,00	0,97
Late Badenian	Karpatian	0,85	0,69
Late Badenian	Early Badenian	0,74	0,33
Late Badenian	Sarmatian	0,99	0,97
Sarmatian	Eggenburgian	0,99	0,99
Sarmatian	Ottnangian	1,00	1,00
Sarmatian	Karpatian	0,98	0,98
Sarmatian	Early Badenian	0,99	0,93
Sarmatian	Late Badenian	0,99	0,97
Early Badenian (Carpathian Basin)	Early Badenian (VB)	0,52	0,49
Early Badenian (Carpathian Basin)	Early Badenian (central basins)	0,58	0,51
Early Badenian (Carpathian Basin)	Early Badenian (southern basins)	0,60	0,50
Early Badenian (VB)	Early Badenian (Carpathian Basin)	0,52	0,49
Early Badenian (VB)	Early Badenian (central basins)	0,52	0,46
Early Badenian (VB)	Early Badenian (southern basins)	0,54	0,46
Early Badenian (central basins)	Early Badenian (Carpathian Basin)	0,58	0,51
Early Badenian (central basins)	Early Badenian (VB)	0,52	0,46
Early Badenian (central basins)	Early Badenian (southern basins)	0,53	0,50
Early Badenian (southern basins)	Early Badenian (Carpathian Basin)	0,60	0,50
Early Badenian (southern basins)	Early Badenian (VB)	0,54	0,46
Early Badenian (southern basins)	Early Badenian (central basins)	0,53	0,50
Late Badenian (southern basins)	Late Badenian (central basins)	0,85	0,84
Late Badenian (southern basins)	Late Badenian (VB)	0,79	0,64
Late Badenian (southern basins)	Late Badenian (Carpathian Basin)	0,87	0,78
Late Badenian (central basins)	Late Badenian (southern basins)	0,85	0,84
Late Badenian (central basins)	Late Badenian (VB)	0,83	0,70
Late Badenian (central basins)	Late Badenian (Carpathian Basin)	0,90	0,82
Late Badenian (VB)	Late Badenian (southern basins)	0,79	0,64
Late Badenian (VB)	Late Badenian (central basins)	0,83	0,70
Late Badenian (VB)	Late Badenian (Carpathian Basin)	0,80	0,79
Late Badenian (Carpathian Basin)	Late Badenian (southern basins)	0,87	0,78
Late Badenian (Carpathian Basin)	Late Badenian (central basins)	0,90	0,82
Late Badenian (Carpathian Basin)	Late Badenian (VB)	0,80	0,79

Multiple-site dissimilarity and turnover

Time interval	Beta div. total	Turnover comp.
Early Badenian	0,671	0,616
Late Badenian	0,878	0,824
